# Long‐lived hypopituitary Ames dwarf mice are resistant to the detrimental effects of high‐fat diet on metabolic function and energy expenditure

**DOI:** 10.1111/acel.12467

**Published:** 2016-03-17

**Authors:** Cristal M. Hill, Yimin Fang, Johanna G. Miquet, Liou Y. Sun, Michal M. Masternak, Andrzej Bartke

**Affiliations:** ^1^ Department of Medical Microbiology, Immunology and Cell BiologySouthern Illinois University School of MedicineSpringfieldILUSA; ^2^ Department of Internal Medicine, Geriatrics ResearchSouthern Illinois University School of MedicineSpringfieldILUSA; ^3^ Faculty of Pharmacy and BiochemistryInstitute of Chemical and Biological Physical ChemistryBuenos AiresArgentina; ^4^ Department of BiologyUniversity of Alabama at BirminghamBirminghamALUSA; ^5^ Burnett School of Biomedical Sciences‐ College of MedicineUniversity of Central FloridaOrlandoFLUSA

**Keywords:** aging, growth hormone, high fat diet, insulin sensitivity, metabolic phenotype

## Abstract

Growth hormone (GH) signaling stimulates the production of IGF‐1; however, increased GH signaling may induce insulin resistance and can reduce life expectancy in both mice and humans. Interestingly, disruption of GH signaling by reducing plasma GH levels significantly improves health span and extends lifespan in mice, as observed in Ames dwarf mice. In addition, these mice have increased adiposity, yet are more insulin sensitive compared to control mice. Metabolic stressors such as high‐fat diet (HFD) promote obesity and may alter longevity through the GH signaling pathway. Therefore, our objective was to investigate the effects of a HFD (metabolic stressor) on genetic mechanisms that regulate metabolism during aging. We show that Ames dwarf mice fed HFD for 12 weeks had an increase in subcutaneous and visceral adiposity as a result of diet‐induced obesity, yet are more insulin sensitive and have higher levels of adiponectin compared to control mice fed HFD. Furthermore, energy expenditure was higher in Ames dwarf mice fed HFD than in control mice fed HFD. Additionally, we show that transplant of epididymal white adipose tissue (eWAT) from Ames dwarf mice fed HFD into control mice fed HFD improves their insulin sensitivity. We conclude that Ames dwarf mice are resistant to the detrimental metabolic effects of HFD and that visceral adipose tissue of Ames dwarf mice improves insulin sensitivity in control mice fed HFD.

## Introduction

According to the Center for Disease Control 2010 Adult Obesity report, the epidemic of obesity contributes to health disparities resulting in higher medical attention and increased pharmaceutical needs and expenses in both middle‐income and low‐income populations in the United States (CDC, 2010). Individuals that are overweight or obese have greater risk of developing chronic diseases such as type 2 diabetes, cardiovascular disease (Kopelman, 2000), and cancer (Calle *et al*., 2003). In addition, these chronic diseases are strongly associated with older age and have negative impacts on longevity (Copaci *et al*., 2015; Sirbu *et al*., 2015).

Mammalian survival depends on balanced nutrient consumption and the ability to fight infection, among other factors. Hormones such as growth hormone (GH) can function in both metabolic and immune roles. Growth hormone targets all tissues, although GH stimulates the production of insulin‐like growth factor 1 (IGF‐1) mainly in the liver (Isaksson *et al*., 1987). Abnormal high levels of GH can contribute to the induction of insulin resistance (Weaver *et al*., 1995), alter inflammatory cytokine levels (Uronen‐Hansson *et. al*., 2003; Bartke, 2008), and can reduce life expectancy in both mice (Brown‐Borg *et al*., 1996; Bartke *et al*., 1998) and humans (Chertman *et al*., 2000). Disruption of GH signaling by either reducing plasma GH levels or by GH receptor deletion significantly extends lifespan in mice as reported in Ames dwarf mice, Snell dwarf mice, GH receptor/GH‐binding protein gene knockout mice (GHR‐KO mice), and GH‐releasing hormone knockout mice (GHRH‐KO) (Brown‐Borg *et al*., 1996; Coschigano *et al*., 2000; Bartke *et al*., 2011). Longevity may also be altered by metabolic stressors such as increased calorie consumption from diets high in fat.

Excessive consumption of calories from diets high in saturated fat have been reported to induce obesity and promote local and systemic inflammation with consequent changes leading to impaired insulin signaling in mice (Roberts‐Toler *et al*., 2015) and humans (Wiedemann *et al*., 2013). This response is also recognized as promotion of obesity‐linked inflammatory diseases including insulin resistance, nonalcoholic fatty liver disease (NAFLD), and atherosclerosis (Sethi & Hotamisligil, 1999). Obesity increases levels of inflammatory adipokines such as interleukin‐6 (IL‐6) and tumor necrosis factor‐alpha (TNF‐α), which induce insulin resistance (Sethi & Hotamisligil, 1999; Fasshauer *et al*., 2003). IL‐6 is elevated by high insulin levels and leads to an increase of TNF‐α in plasma and white adipose tissue, which negatively impacts insulin signaling by inducing serine phosphorylation of the insulin receptor substrate‐1 (IRS‐1). Serine phosphorylation of IRS‐1 inhibits association with the insulin receptor stimulating degradation of IRS‐1 (Fasshauer *et al*., 2003). Moreover, anti‐inflammatory adipokines such as insulin sensitizer adiponectin are decreased by excess weight gain and this decrease promotes insulin resistance in mice (Yamauchi *et al*., 2001) and humans (Tschritter *et al*., 2003; Altinova *et al*., 2007).

Long‐lived hypopituitary Ames dwarf mice and GH‐resistant GHR‐KO mice share various metabolic characteristics such as suppressed levels of circulating IGF‐1, insulin, and glucose and are more insulin sensitive, yet have increased adiposity compared to their control littermates (Berryman *et al*., 2004; Bartke, 2008; and Bartke *et al*., 2011). The visceral adipose tissue in Ames dwarf and GHR‐KO mice has been reported to benefit their metabolic profile, which is related to their extended longevity. Enhanced insulin sensitivity in these animals was related to higher levels of adiponectin in comparison with their control (normal) littermates fed standard diet. Furthermore, Ames dwarf and GHR‐KO mice have reduced respiratory quotient, indicating increased reliance on fat instead of carbohydrate as metabolic fuel, which has been associated with their improved metabolic function (Westbrook *et al*. 2009).

Although Ames dwarf mice have improved health span compared to control mice fed normal chow, the impact of high‐fat diet (HFD) on the metabolic pathways and metabolic profiles in long‐lived Ames dwarf mice remains to be shown. Therefore, we decided to investigate the effects of HFD as a metabolic stressor in long‐lived Ames dwarf mice to assess the interaction of diet and genetic mechanisms that regulate metabolism and pathogenesis during aging. The objectives of our study was to test whether animals that have increased lifespan and altered endocrine signaling are adversely impacted by a HFD and whether transplant of visceral adipose tissue from Ames dwarf mice fed HFD into control mice fed HFD would alter insulin sensitivity in the recipient mice.

## Results

### Ames dwarf mice have greater adiposity gain in response to HFD feeding

**Figure 1:**
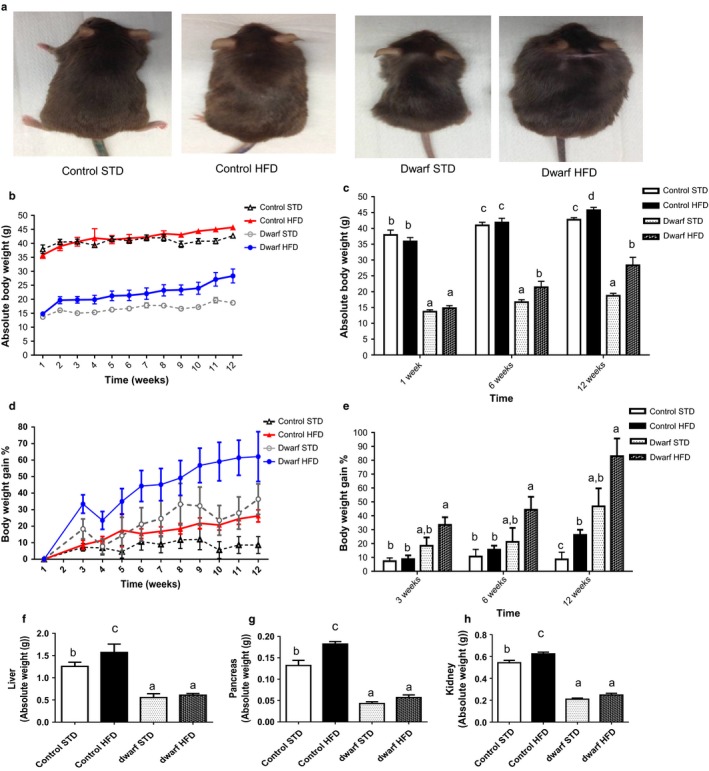
Ames dwarf mice are sensitive to diet‐induced obesity. (a) Ames dwarf and control mice consumed either an STD or HFD for 12 weeks. Weekly body weights were recorded during the feeding phase (*n *=* *10 per group). (b) Absolute body weight. (c) Absolute body weight at week 1, week 6, and week 12. (d) Body weight gain percentage. (e) Body weight gain percentage at week 3, week 6, and week 12. (f) Absolute liver weight. (g) Absolute pancreas weight. (h) Absolute kidney weight. All values are presented as mean. The error bar represents the SEM. Groups that do not share a superscript are different with statistical significance (*P* < 0.05).

To assess the effects of diet‐induced obesity in mice that are GH‐deficient on weight gain and adipose tissue, Ames dwarf and control mice were fed either HFD or standard diet (STD) for 12 weeks. In both Ames dwarf and control mice, HFD feeding produced an increase in body weight compared to their counterparts fed STD (Fig. 1a and b). After 1 week of feeding, there was no difference between same phenotypes fed either HFD or STD (Fig. 1c). However, at 6 weeks, only Ames dwarf mice fed HFD had increased body weights compared to Ames dwarf mice fed STD (*P *< 0.04) (Fig. 1c). At 12 weeks, both Ames dwarf mice (*P* < 0.003) and control mice (*P* < 0.01) fed HFD had increased in body weight compared to their counterparts fed STD. Body weight gain during the 12 week feeding period was approximately 15% greater in control mice on HFD than control mice fed STD (Fig. 1d). After 12 weeks, in Ames dwarf mice fed HFD body weight gain was approximately 30% greater than in Ames dwarfs fed STD and 40% greater than in control mice fed either STD or HFD (Fig. 1d). Particularly, after 3 weeks of acclamation to diet, Ames dwarf mice fed HFD had a greater increase in weight gain compared to control mice fed HFD (*P* < 0.001) (Fig. 1e). However, there was no difference in body weight gain in Ames dwarf fed HFD compared to Ames dwarf mice fed STD (*P* = 0.08) (Fig. 1e). Furthermore, there was no difference in weight gain in control mice fed HFD compared to control mice fed STD (*P* = 0.69) (Fig. 1e). At 6 weeks, this same trend was observed in Ames dwarf mice fed HFD compared to either Ames dwarf mice fed STD or control mice fed either STD or HFD (Fig. 1e). At 12 weeks, during the final feeding phase, Ames dwarf mice fed HFD increased in body weight gain compared to control mice fed HFD (*P* < 0.0006), yet this seeming increase was not different compared to Ames dwarf mice fed STD (*P* = 0.07) (Fig. 1e). As expected from HFD feeding, control mice fed HFD increased in body weight gain compared to control mice fed STD (*P* < 0.01) (Fig. 1e). In addition, Ames dwarf fed STD increased in body weight gain compared to control mice fed the same diet (*P* < 0.01) (Fig. 1e). These data suggest that GH‐deficient Ames dwarf mice respond to induction of obesity by HFD feeding greater than control mice fed the same diet.

As Ames dwarf mice fed HFD greatly increased in percentage of whole body weight gain compared to control mice fed either HFD or STD, we next examined if the effects of HFD would alter organ weights in Ames dwarf and control mice. Liver weight was significantly increased in control mice fed HFD compared to control mice fed STD and Ames dwarf mice fed either HFD or STD (*P* < 0.03) (Fig. 1f) possibly reflecting increased lipid deposits in peripheral tissues (Marceau *et al*., 1999). The same trend was also observed in pancreas weight (*P* < 0.02) (Fig. 1g). Furthermore, kidney weight was increased in control mice fed HFD compared to control mice fed STD (*P* < 0.009). Yet, HFD feeding did not alter liver, pancreas, or kidney weight in Ames dwarf fed HFD compared to Ames dwarf fed STD. There were no differences in heart or whole brain weights as an effect of diet within either Ames dwarf or control mice (*data not shown*).

**Figure 2:**
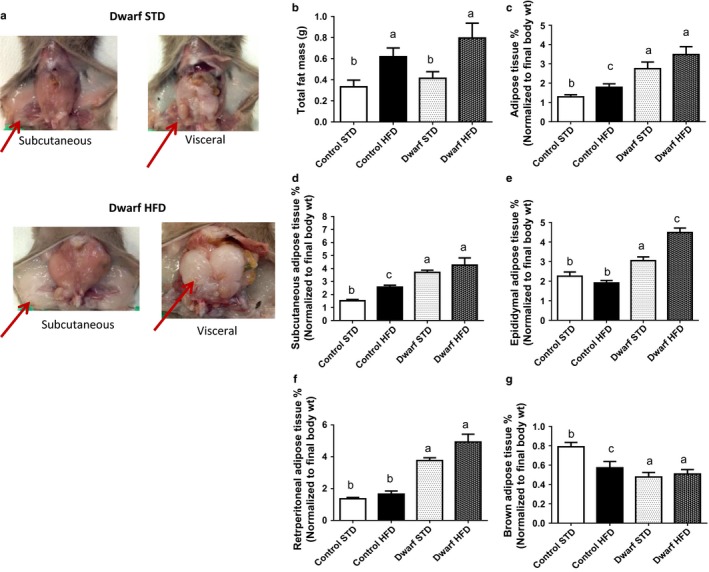
Visceral fat is increased in Ames dwarf mice fed HFD diet. (a) Abdominal cavity terminal dissection of subcutaneous and visceral adipose tissue in Ames dwarf fed either STD or HFD. (b) Absolute fat mass (subcutaneous, epididymal, retroperitoneal, and brown). (c) Normalized total adipose tissue. (d) Normalized subcutaneous adipose tissue. (e) Normalized epididymal adipose tissue. (f) Normalized retroperitoneal adipose tissue. (g) Normalized interscapular brown adipose tissue (iBAT). All values are presented as mean. The error bar represents the SEM. Groups that do not share a superscript are different with statistical significance (*P* < 0.05).

To further examine the interaction of HFD feeding and GH signaling, we questioned whether adipose tissue weights would be altered in Ames dwarf and control mice fed either HFD or STD. Data obtained from necropsy indicated that Ames dwarf mice fed HFD for 12 weeks greatly increased their subcutaneous white adipose tissue (WAT) and visceral WAT (VAT) compared to Ames dwarf mice fed STD (Fig. 2a). Furthermore, absolute total fat mass was increased in both Ames dwarf and control mice fed HFD compared to their counterparts fed STD (*P* < 0.02) (Fig. 2b) (absolute adipose tissue weights are shown in Fig. S2a–d). It is well documented that GH‐resistant mice have more adiposity compared to control mice fed standard chow (Berryman *et al*., 2004), and here, similarly we show that GH‐deficient Ames dwarf mice fed STD have more body fat compared to control mice fed STD. The normalized (percentage) total adipose tissue in relation to total body weight was higher in Ames dwarf mice fed STD compared to control mice fed either STD or HFD (*P* < 0.008), yet Ames dwarf mice fed HFD did not differ from Ames dwarf fed STD (*P* = 0.1810) (Fig. 2c). Furthermore, percentage of adipose tissue increased in control mice fed HFD compared to control mice fed STD (*P* < 0.02) (Fig. 2c). In particular, Ames dwarf mice fed HFD had significantly increased percentage of the subcutaneous (*P* < 0.01), epididymal (*P* < 0.0002), and retroperitoneal (*P* < 0.0003) adipose tissue when compared to control mice fed either STD or HFD (Fig. 2d,e, and f). Moreover, only percentage of epididymal adipose tissue increased in Ames dwarf mice fed HFD compared to Ames dwarf mice fed STD (*P* < 0.001) (Fig. 2e). In contrast, control mice fed HFD increased in percentage of subcutaneous adipose tissue compared to control mice fed STD (*P* < 0.008) (Fig. 2d). There was no difference in percentage of epididymal and retroperitoneal adipose tissue in control mice fed HFD compared to control mice fed STD (Fig. 2e and f). The percentage of interscapular brown adipose tissue (iBAT) in control mice fed HFD decreased compared to control mice fed STD (*P* < 0.02) (Fig. 2g). There was no difference in percentage of iBAT in Ames dwarf mice either STD or HFD (Fig. 2g). These data represent that GH‐deficient Ames dwarf mice, which have naturally increased adiposity, have greater gain in adiposity in epididymal adipose tissue when fed HFD compared to their counterparts fed STD and control mice fed either STD or HFD. Furthermore, HFD does not change iBAT in Ames dwarf mice fed either diet, yet the percentage of iBAT adipose tissue is significantly reduced in control mice fed HFD compared to control mice fed STD.

### Ames dwarf mice retain insulin sensitivity when challenged with high‐fat diet

**Figure 3:**
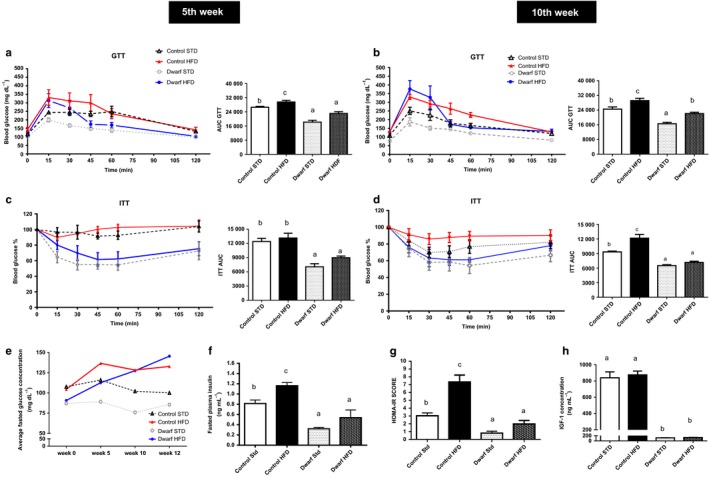
Long‐lived Ames dwarf mice are resistant to metabolic dysfunction induced by HDF diet. Ames dwarf and control mice consumed either STD or HFD diet for 12 weeks; GTT and ITT were performed during week 5 and week 10 of the study (*n *=* *10 per group). (a) and (b) Glucose tolerance test (GTT) and area under the curve at 5 weeks and 10 weeks. Sixteen‐hour‐fasted mice underwent GTT by i.p. injection with 1 g glucose per kg of BW. (c) and (d) Insulin tolerance test (ITT) and area under the curve at 5 weeks and 10 weeks. Mice were injected i.p. with 1.0 IU porcine insulin per kg of BW. ITT data expressed as percentage of initial blood glucose concentration. (e) Average blood glucose levels. (f) Fasted insulin levels (g) HOMA‐IR Score (h) Circulating IGF‐1 levels. All values are presented as mean. The error bars represent the SEM. Groups that do not share a superscript are different with statistical significance (*P* < 0.05).

An increase in weight gain, specifically in visceral adipose tissue, is associated with increased risks of metabolic dysfunction, such as insulin resistance. We show that Ames dwarf fed HFD increased in epididymal adipose tissue compared to Ames fed STD; therefore, we next evaluated glucose homeostasis in Ames dwarf and control mice fed either HFD or STD by glucose tolerance testing (GTT) and insulin tolerance testing (ITT). As revealed by GTT, Ames dwarf mice fed a HFD at the intermediate phase (5 weeks) of the study exhibited a rate of reduction in the clearance of glucose from the blood at 15 and 30 min after glucose injection (Fig. 3a). However, Ames dwarf mice were able to clear blood glucose at 45, 60, and up to 120 min better than the control mice fed HFD as shown by area under the curve (*P* < 0.001) (Fig. 3a). In addition, Ames dwarf fed HFD were more glucose tolerant compared to control mice fed STD (*P* < 0.007). Furthermore, glucose tolerance in Ames dwarf mice fed HFD did not differ from Ames dwarf mice fed STD as shown by area under the curve (*P* = 0.22) (Fig. 3a). The pattern of blood glucose clearance seen at week 5 was also observed at the final phase (week 10) of this study, when Ames dwarf mice fed HFD were more glucose tolerant than control mice fed HFD as shown by area under the curve (*P* < 0.0001) and resembled control mice fed STD (Fig. 3b) (*P* = 0.30). Previous reports have shown that Ames dwarf mice are more insulin sensitive compared to control mice when they are fed STD (Bartke, 2008; and Bartke *et al*., 2011). As expected, Ames dwarf mice fed STD were more insulin sensitive compared to control mice fed STD as shown by area under the curve (*P* < 0.004) (Fig. 3c). Surprisingly, Ames dwarf mice fed HFD were more insulin sensitive compared to control mice fed either HFD or STD at the intermediate (week 5) phase of the study as shown by area under the curve (*P* < 0.001) (Fig. 3c). Moreover, insulin sensitivity was not altered in Ames dwarfs fed HFD compared to Ames dwarfs fed STD (*P* = 0.06) (Fig. 3c). There was no difference in insulin sensitivity in control mice fed either STD or HFD at 5 weeks (Fig. 3c). Similar to insulin sensitivity observed at week 5 in Ames dwarf mice fed HFD compared to Ames dwarf mice fed STD, there was no difference in insulin sensitivity in these groups at week 10 (*P* = 0.08). This remarkable difference between Ames dwarf and control mice was maintained and observed at the final phase (week 10) of the study when Ames dwarf mice fed HFD were more insulin sensitive than control mice fed HFD as shown by area under the curve (*P* < 0.007) (Fig. 3d). Furthermore, control mice fed HFD had reduced insulin sensitivity compared to control mice fed STD as shown by area under the curve (*P* < 0.007) (Fig. 3d). These findings indicate that Ames dwarf mice fed a HFD retained their enhanced insulin sensitivity similarly to Ames dwarf fed STD when compared to control mice fed HFD, thus exhibiting resistance to the induction of endocrine dysfunction by HFD feeding.

As an expected effect of a saturated fat diet, fasted blood glucose levels were elevated in both Ames dwarf and control mice fed HFD compared to their counterparts fed STD (Fig. 3e). Insulin levels were elevated in control mice fed HFD compared to control mice fed STD (*P* < 0.009), yet in Ames dwarf mice fed HFD, insulin levels remained low compared to control mice fed HFD diet (*P* < 0.002) (Fig. 3f). Moreover, the apparent increase in insulin levels in Ames dwarf mice fed HFD was not statistically significant compared to Ames dwarf mice fed STD (*P* = 0.08) (Fig. 3f). Homeostasis model assessment‐insulin resistance (HOMA‐IR) risk scores, which are used to quantify insulin resistance and beta cell function, were lower in Ames dwarf mice fed HFD than in control mice fed HFD (*P* < 0.0002) (Fig. 3g). Furthermore, control mice fed HFD had a higher HOMA‐IR score when compared to control mice fed STD (*P* < 0.002) (Fig. 3g). Ames dwarf mice fed STD had a lower HOMA‐IR risk score when compared to control mice fed STD (*P* < 0.001) (Fig. 3g). There was no difference in HOMA‐IR score between Ames dwarf mice fed HFD when compared to Ames dwarf mice fed STD (*P* = 0.10). Apparently, Ames dwarf mice fed STD are at low risk for metabolic dysfunction compared to control mice fed STD. These data indicate that HFD feeding does not increase the risk of metabolic dysfunction in Ames dwarf, in contrast to control mice.

Previous studies have reported that IGF‐1 can mimic the metabolic effects of insulin to stimulate glucose uptake (Blundell & Humbel, 1980; Di Cola *et al*., 1997). To further clarify endocrine function, we evaluated IGF‐1 levels in Ames dwarf and control mice fed either diets. Ames dwarf mice fed STD typically have undetectable levels of IGF‐1 (Bartke, 2008). We observed the expected decrease of systemic IGF‐1 levels in Ames dwarf compared to control mice (*P* < 0.0001) and there was no difference IGF‐1 levels in Ames dwarf or control mice fed HFD compared to their counterparts fed STD (Fig. 3h).

### Ames dwarf mice fed high‐fat diet are protected from pro‐inflammatory adipokines

**Figure 4:**
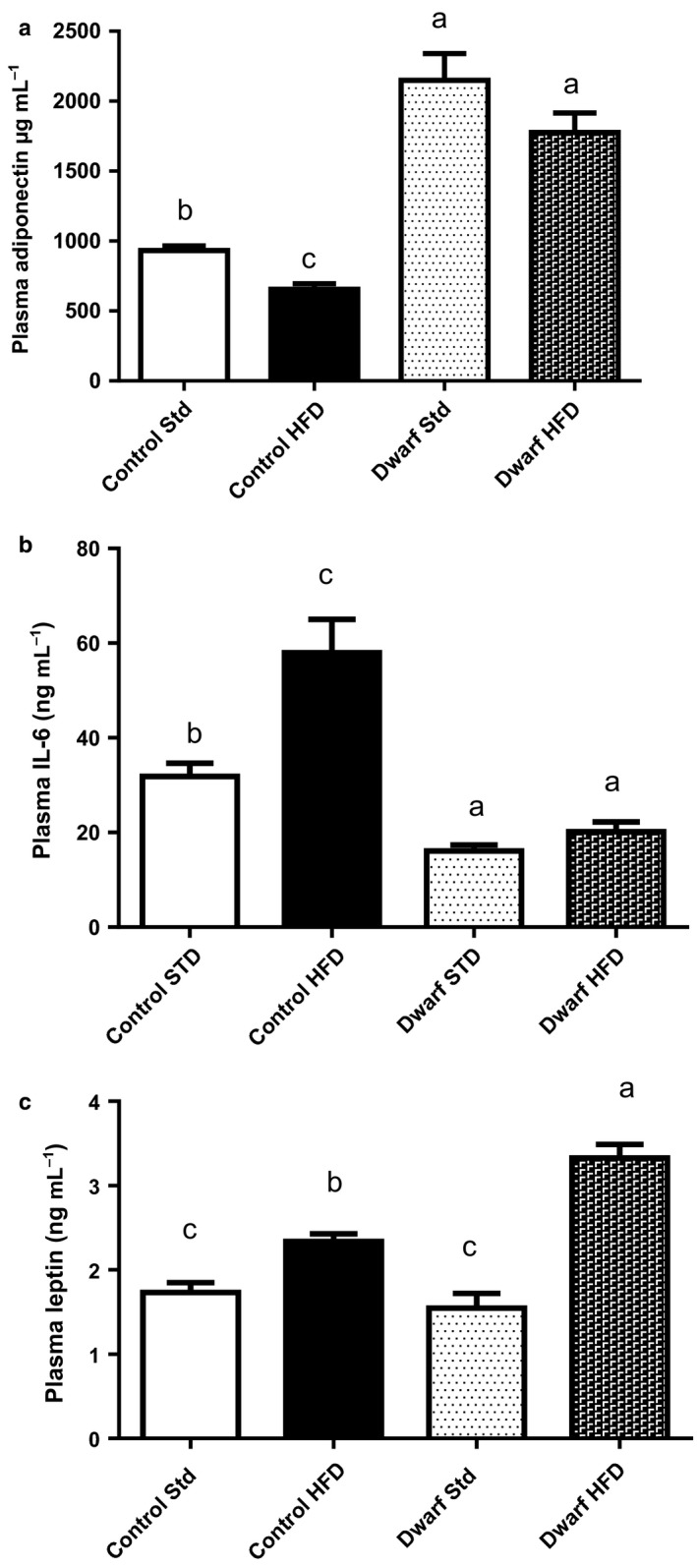
Anti‐inflammatory cytokine levels are increased in Ames dwarf mice fed HFD diet. Mice were fasted for 16 h, and plasma was used for assays (*n *=* *8–10 per group). (a) Mouse Adiponectin. (b) Interleukin 6 (IL‐6). (c) Leptin. All values are presented as mean. The error bars represent the SEM. Groups that do not share a superscript are different with statistical significance (*P* < 0.05).

Alterations in the levels of adipokines including a decrease in adiponectin (Berg *et al*., 2002) and increases in leptin (Wauman & Tavernier, 2011) and IL‐6 (Vozarova *et al*., 2001) are primary links between obesity and systemic inflammation, as well as signs of progression of metabolic dysfunction. In contrast to the generally observed reciprocal relationship between obesity and adiponectin levels, Ames dwarf mice fed a HFD retained high levels of adiponectin when compared to control mice fed HFD (*P* < 0.0001) and did not differ from Ames dwarf mice fed STD (*P* = 0.15) (Fig. 4a). However, control mice fed HFD had decreased adiponectin levels compared to control mice fed STD (*P* < 0.002) (Fig. 4a). In addition, Ames dwarf mice fed HFD had lower levels of IL‐6 than control mice fed either diet (*P* < 0.007) and did not differ from Ames dwarf mice fed STD (*P* = 0.12). Moreover, control mice fed HFD had higher levels of IL‐6 compared to control mice fed STD (*P* < 0.003) (Fig. 4b). These data suggest that Ames dwarf mice fed HFD have higher levels of insulin sensitizer adiponectin and lower systemic levels of IL‐6 which is associated with their ability to be more insulin sensitive as revealed by ITT compared to control mice fed HFD. Plasma leptin levels were significantly increased in Ames dwarf mice fed HFD compared to Ames dwarf fed STD (*P* < 0.004) and control mice fed either STD (*P* < 0.0001) or HFD (*P* < 0.008), corresponding to the observed changes in adiposity (Fig. 4c). In addition, leptin levels were higher in control mice HFD compared to control mice fed STD (*P* < 0.002) (Fig. 4c).

### High‐fat diet impacts lipid levels and energy expenditure in Ames dwarf mice

**Figure 5:**
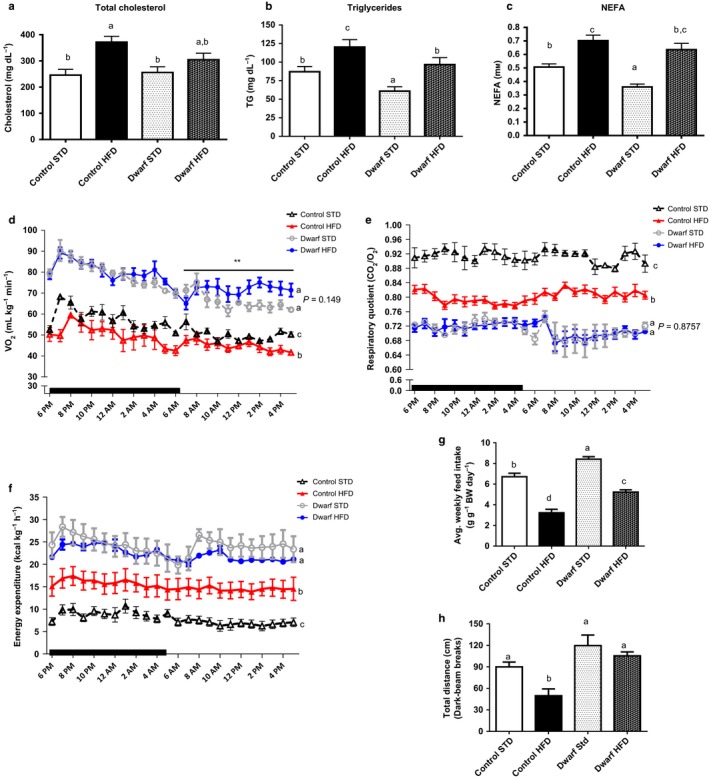
Ames dwarf mice fed HFD have increased VO2, decreased RQ, and enhanced energy expenditure. Ames dwarf mice (*n *=* *6) and control mice (*n *=* *6) fed STD and Ames dwarf mice (*n *= 6) and control mice (*n *= 6) fed HFD were placed in the metabolic chambers to measure VO2, RQ and locomoter activity. At 12 weeks, mice were fasted for 16 h, and plasma was used for (a) Total Cholesterol. (b) Triglycerides. (c) Non‐ esterified free fatty acids. (d) VO2 (e) RQ (f) Calculated energy expenditure. (g) Food consumption (h) Locomoter activity. All values are presented as mean. The error bars represent the SEM. Groups that do not share a superscript are different with statistical significance (*P* < 0.05).

Increased levels of cholesterol, triglycerides, and nonesterified fatty acids are typically observed in obese patients. Also, dysregulation of lipid metabolism has been identified as a critical contributor to the mechanistic etiology of insulin resistance (Ginsberg *et al*., 2006). Control mice fed HFD had increased total plasma cholesterol levels when compared to control and Ames dwarf mice fed STD (*P* < 0.003) (Fig. 5a). Furthermore, the level of total cholesterol appeared slightly increased in Ames dwarf mice fed HFD compared to Ames dwarf mice fed STD, but this apparent difference was not statistically significant (*P* = 0.17) (Fig. 5a). There was no difference in cholesterol levels in Ames dwarf mice fed STD compared to control mice fed STD (*P* = 0.75). Control mice fed HFD had significantly increased triglyceride levels compared to control mice fed STD (*P* < 0.004), and a similar trend was observed in Ames dwarf mice fed HFD compared to Ames dwarf mice fed STD (*P* < 0.003) (Fig. 5b). However, peripheral triglyceride levels were lower in Ames dwarf mice fed HFD compared to control mice fed HFD (*P* < 0.001), while they did not differ from the value measured in control mice fed STD (*P* = 0.57). Nonesterified fatty acid (NEFA) levels were significantly increased in control mice fed HFD compared to control mice fed STD (*P* < 0.001) (Fig. 5c). This was also observed in Ames dwarf mice fed HFD when compared to Ames dwarf mice fed STD (*P* < 0.0003). Furthermore, Ames dwarf mice fed STD had lower levels of NEFAs when compared to control mice fed STD (*P* < 0.0008) (Fig. 5c). There was no difference in NEFA levels in Ames dwarf mice fed HFD compared to control mice fed HFD (*P* = 0.31).

Ames dwarf mice are sensitive to weight gain and increase in triglycerides and NEFA's in response to HFD feeding compared to their counterparts fed STD, yet are resistant to the induction of metabolic dysfunction by HFD feeding as revealed by ITT and GTT and they retain elevated adiponectin levels. Therefore, we questioned whether their energy metabolism profile would expound on their resistance to metabolic stressors such as HFD. We approached this question using indirect calorimetry to monitor respiratory quotient, oxygen consumption, and locomotor activity and to calculate energy expenditure in Ames dwarf and control mice fed either HFD or STD during the 10th week of the feeding phase.

Oxygen consumption (VO_2_) per unit body mass was significantly higher in Ames dwarf mice fed either diet than in control mice on STD. Ames dwarf mice fed STD consumed more oxygen than control mice fed STD (Fig. S3a; *P* < 0.0001). These data are consistent with our previous findings that Ames dwarf consume more oxygen per unit body mass than control mice when they are fed standard chow (Westbrook *et al*. 2009). At 10 weeks of HFD feeding, Ames dwarf mice showed differences in VO_2_ compared to Ames dwarf mice fed STD with a remarkable increase in the amount of oxygen consumed during the light hours (*P* < 0.03) (Fig. 5d). However, there was no difference in the average VO_2_ per 24 h in Ames dwarf mice fed HFD compared to Ames dwarf mice fed STD (*P* = 0.149) (Fig. 5d). In addition, Ames dwarf mice fed HFD consumed more oxygen than control mice fed HFD (Fig. S3b *P* < 0.0001); and control mice fed HFD consumed less oxygen than control mice fed STD (Figs 5d and S3c; *P* < 0.002). These data show that Ames dwarf mice fed HFD retain their increased VO2during both their active hours (dark) and during their inactive hours (light) when compared to control mice fed either HFD or STD.

Having a lower respiratory quotient (RQ) value indicates increased lipid usage as main source of energy in mammalian energy homeostasis (Lusk, 1928). We have shown that Ames dwarf mice fed HFD have higher levels of NEFAs, similar to control mice fed HFD, than Ames dwarf fed STD. At 10 weeks, RQ values were significantly lower in Ames dwarf fed HFD when compared to control mice fed HFD during the 24‐h measurement (Fig. 5e) (Fig. S3d; *P* < 0.0001). However, there were no differences on RQ between Ames dwarf mice fed HFD and those fed STD (*P* = 0.8757) (Fig. 5e) (Fig. S3d). As expected, control mice fed HFD had lower RQ value than control mice fed STD (*P* < 0.001) (Fig. 5e) (Fig. S3d; *P* < 0.001). Furthermore, Ames dwarf mice fed STD had a lower RQ when compared to control mice fed STD (Fig. 5e) (Fig. S3d; *P* < 0.0001), which is consistent with previous data from our laboratory (Westbrook *et al*., 2009). As indicated by RQ values, these data show that HFD fed Ames dwarf mice retain the ability to rely on lipid as their main source of energy greater than control mice fed HFD.

At 10 weeks, Ames dwarf mice fed HFD had higher energy expenditure per unit of body mass compared to control mice fed HFD (*P* < 0.0001) (Fig. 5f) (Fig. S3e; *P* < 0.0001). Control mice fed HFD had significantly higher energy expenditure than control mice fed STD (Fig. 5f) (Fig. S3e; *P* < 0.0001). Corresponding to our previous data that Ames dwarf mice fed STD produce more heat per gram of body weight (Westbrook *et al*., 2009), we observed that energy expenditure was higher in Ames dwarf fed STD compared to control mice fed STD (Fig. 5f) and (Fig. S3e; *P* < 0.0001).

Our laboratory previously reported that Ames dwarf mice of both sexes consume more food per gram of body weight than control mice when fed standard chow (Mattison *et al*., 2000). However, whether food intake in Ames dwarfs consuming HFD is also altered was unknown. In this study, we observed that food intake was decreased in both Ames dwarf (*P* < 0.0001) and control (*P* < 0.0001) mice fed HFD compared to their counterparts fed STD (Fig. 5g), and this could be a result of the increased leptin levels observed in both phenotypes when fed HFD. We also observed the expected increase in food intake in Ames dwarf mice fed STD compared to control mice fed STD (*P* < 0.002) (Fig. 5g).

Spontaneous locomotor activity as measured by distance traveled was greater in Ames dwarf mice fed HFD compared to normal mice fed HFD during dark hours, their most active time (*P* < 0.002) (Fig. 5h). Interestingly, there was no difference in Ames dwarf mice when fed either HFD or STD (*P* = 0.398) while HFD feeding in control mice decreased locomotor activity (*P* < 0.01) (Fig. 5h). There were no locomotor activity differences observed during light hours in control or dwarf mice fed either diet (Fig. S3f).

### Transplant of visceral adipose tissue of Ames dwarf mice fed HFD improves insulin sensitivity in control mice fed HFD

**Figure 6:**
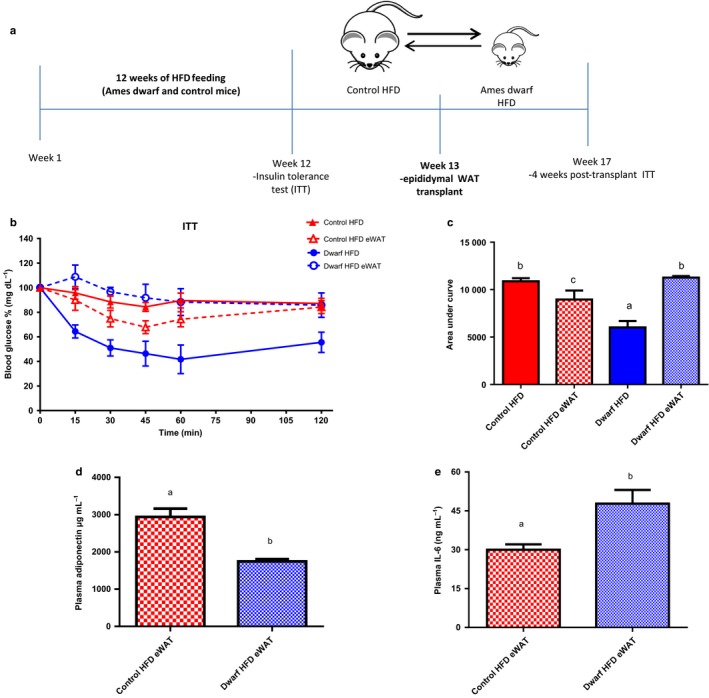
Transplant of visceral adipose tissue of Ames dwarf rescues insulin sensitivity in control mice fed HFD. Ames dwarf (*n *=* *4) and control mice (*n *=* *4) fed a HFD for 12 weeks; Insulin tolerance test (ITT) was performed at the 12 week prior to eWAT transplant. 4 weeks post‐ eWAT transplant ITT was performed in Ames dwarf and control mice fed HFD. Mice were injected i.p. with 1.0 IU porcine insulin per kg of BW. ITT data expressed as percentage of initial blood glucose concentration. (a) eWAT transplant summary diagram; interchange of epididymal white adipose tissue between Ames dwarf and control mice fed HFD (b) ITT prior (solid lines) and after (dash lines) eWAT. (c) Area under curve of ITT. Mice were fasted for 16 h, and plasma was used for assays. (d) Mouse Adiponectin. (f) Interleukin 6 (IL‐6). eWAT: epididymal white adipose tissue. All values are presented as mean. The error bars represent the SEM. Groups that do not share a superscript are different with statistical significance (*P* < 0.05).

We have shown that Ames dwarf mice fed HFD are resistant to the detrimental effects of a HFD (60% kcal from fat), as revealed by persistence of increased insulin sensitivity, increased adiponectin levels, and reduced respiratory quotient. Previous studies have indicated that long‐lived mice that either are GH‐deficient or exhibit disrupted GH signaling have increased adiposity and systemic high levels of adiponectin (Berryman *et al*., 2004; Wang *et al*., 2006). The removal of epididymal visceral fat decreased levels of adiponectin and reduced insulin sensitivity in GHR‐KO and Ames dwarf mice (Masternak *et al*., 2012; Menon *et al*., 2014). In the context that in the absence of GH signaling, visceral fat exerts unexpected beneficial influence on insulin sensitivity (Bartke, 2008), and in this study Ames dwarf mice are resistant to the detrimental effects of HFD, we were interested in determining whether transplantation of visceral fat from Ames dwarf mice fed HFD into control littermates fed the same diet could improve their insulin sensitivity. In this experiment, we fed Ames dwarf mice and control mice HFD for 12 weeks and accessed their insulin sensitivity by ITT. During the 13th week of HFD feeding, we surgically exchanged epididymal WAT (eWAT) between Ames dwarf and their control siblings. After transplant surgeries, mice continued consuming HFD for an additional period of 4 weeks and insulin sensitivity was reassessed by ITT at week 17 (Fig. 6a).

As expected from the results of our earlier ITTs, after 12 weeks of HFD, Ames dwarf mice fed HFD retained their insulin sensitivity compared to control mice fed HFD (Fig. 6b) as shown by area under the curve (*P* < 0.006) (Fig. 6c). At week 17 (4 weeks post‐transplant), insulin sensitivity, as reassessed by i.p. ITT, was significantly improved in the control mice fed HFD that received eWAT from Ames dwarf mice fed HFD (control HFD eWAT) compared to their pretransplant values (control HFD) (Fig. 6b), as shown by area under the curve (*P* < 0.03) (Fig. 6c). Moreover, insulin sensitivity was significantly decreased in Ames dwarf mice fed HFD that received eWAT from control mice fed HFD (dwarf HFD eWAT mice) compared to their pretransplant values (dwarf HFD) (Fig. 6b), as shown by area under the curve analysis (*P* < 0.0002) (Fig. 6c). Four weeks after the exchange of the epididymal fat pads, insulin sensitivity was lower in dwarf HFD eWAT mice compared to control HFD eWAT mice (*P* < 0.01) (Fig. 6c). We further questioned if the levels of adiponectin and IL‐6 would be altered in these same animals after the eWAT transplant surgeries. Adiponectin levels were significantly higher in control HFD eWAT mice compared to dwarf HFD eWAT mice (*P* < 0.001) (Fig. 6d). In addition, IL‐6 levels were lower in control HFD eWAT mice compared to dwarf HFD eWAT mice (*P* < 0.01) (Fig. 6e).

These data complement the reports that the removal of both epididymal and perinephric WAT reduces insulin sensitivity in Ames dwarf (Menon *et al*., 2014) and GHR‐KO mice (Masternak *et al*., 2012) and suggest that the eWAT transplant from long‐lived mice improves metabolic function in control mice fed HFD hence aids in the protection against the effects of HFD‐induced metabolic dysfunction.

## Discussion

The findings in this study indicate that Ames dwarf mice are resistant to the detrimental effects of diet‐induced obesity on metabolic function and have the ability to efficiently utilize fat as their main energy source. Remarkably, greater body weight gain, higher visceral adiposity, and increased NEFAs in Ames dwarf males fed HFD do not induce insulin resistance nor alter their energy homeostasis. We first confirm three major strong associations that may represent mechanisms of longevity in mammals: the relationship of adiponectin to insulin sensitivity, association of leptin signaling with energy homeostasis, and the relationship between enhanced energy expenditure and extended longevity. Second, we suggest that higher adiponectin‐producing adipose tissues from Ames dwarf mice fed HFD are able to improve glucose homeostasis in control mice fed HFD.

### Adiponectin and insulin sensitivity

The cellular mechanism by which adiponectin influences insulin sensitivity is not fully understood, yet the association of adiponectin levels with insulin sensitivity has been extensively reported (Werner *et al*., 2004). Adiponectin levels are more abundant in plasma of lean human subjects (Coppack, 2005). In contrast, levels of adiponectin are decreased in obese human subjects promoting the onset of metabolic syndrome (Tschritter *et al*., 2003; Altinova *et al*., 2007). Recent *in vivo* studies have uncovered beneficial effects of adiponectin treatment on metabolic function. In male mice fed HFD, intracerebroventricular administration of adiponectin led to improved glucose homeostasis and reduced inflammatory signaling (Koch *et al*., 2014). The increase in adiponectin levels has been well established in long‐lived mice such as Ames dwarf (Wang *et al*., 2006), Snell dwarf (Brooks *et al*., 2007) and GHR‐KO (Berryman *et al*., 2004) mice. In the present study, circulating adiponectin levels were significantly higher in Ames dwarf fed HFD when compared to control mice on either diet. In addition, absolute levels of adiponectin were slightly lower in Ames dwarf mice fed HFD, when compared to same phenotype fed STD, but they did not reach significance. We suggest that the increase of adiposity coexisted with the increase of adiponectin levels in Ames dwarf mice because of the increase in size and spacial distribution of adipocytes in these animals as compared to control. Our findings suggest that increased adiponectin levels may have allowed these mice to remain insulin sensitive while consuming HFD or at the very least, they contributed to this state.

### Leptin and glucose homeostasis

Leptin has been reported to mediate important functions in the regulation of neuroendocrine pathways and energy homeostasis (Badman & Flier, 2007). Primarily secreted from adipocytes, leptin levels are proportional to body fat mass (Badman & Flier, 2007), yet leptin gene expression has also been shown to be present at low levels in the liver and skeletal muscle (Meier & Gressner, 2004). Leptin bound to its receptor induces Janus activating kinase/signal transducer and activator of transcription**,** mitogen‐activated protein kinase, phosphatidylinsitol 3′kinase**,** and AMP‐activated protein kinase (AMPK) (Vozarova *et al*., 2001). Activation of the AMPK pathway stimulates glucose uptake (Kurth‐Kraczek *et al*., 1999) and lipid oxidation (Minokoshi *et al*., 2002) to produce energy while simultaneously reduces energy consuming processes (Friedman & Halaas, 1998). This regulation of energy metabolism takes place in multiple peripheral tissues including skeletal muscle, liver, adipose tissues, and pancreatic beta cells which all function in either insulin sensitivity or insulin resistance. Previous studies from our laboratory showed no difference in leptin levels in Ames dwarf mice fed STD compared to control mice fed the same diet or control mice subjected to 30% calorie restriction (CR). However, subjecting Ames dwarf mice to 30% CR significantly increases leptin levels compared to Ames dwarf mice fed STD and control mice fed either diet (Wang *et al*., 2006). In the present study, we observed increased levels of leptin in Ames dwarf mice fed HFD compared to Ames dwarf mice fed STD and control mice fed either diet. These data indicate that higher levels of leptin in Ames dwarf mice fed HFD may possibly stimulated glucose uptake and lipid oxidation, thus balancing glucose homeostasis, as observed in our ITT and GTT data.

### Energy expenditure

Diets such as HFD can alter lipid metabolism and promote metabolic dysfunction by inducing insulin resistance and altering energy expenditure (Ravussin *et al*., 1988). We observed high levels of NEFAs in Ames dwarf mice fed HFD compared to Ames dwarf fed STD and control mice fed either diet. We could only assume that intracellular signaling in peripheral tissues aided the breakdown of triglycerides into NEFAs to be utilized as energy substrates (Minokoshi *et al*., 2002) preserving glucose homeostasis in Ames dwarf mice fed HFD, as observed in our ITT and GTT data.

Oxygen consumption is considered as a gauge of anabolic health in both mice and humans (McGandy *et al*., 1966; Ravussin *et al*., 1988) and is associated to improved health and longevity. Previous studies in our laboratory reported that ‘long‐lived mice’, such as Ames dwarf and GHR‐KO mice (with deficient GH signaling) have increased VO_2_ per unit of body weight in both fed and fasted conditions when compared to their control mice when fed a standard diet (Westbrook *et al*., 2009). In this study, we report that Ames dwarf mice fed HFD had higher VO_2_ levels compared to control mice fed either HFD or STD, yet they did not differ from Ames dwarf mice fed STD. Moreover, control mice fed HFD had lower VO_2_ than control mice fed STD. These data suggest that in Ames dwarf mice oxygen consumption is not affected by HFD feeding, possibly reflecting that an increase in respiration is linked to an increase in fatty acid oxidation (Garcia‐Roves *et al*., 2007; Turner *et al*., 2007).

High RQ values indicate preferential carbohydrate oxidation and are associated with metabolic syndromes such as insulin resistance (Zurlo *et al*., 1990). Previous studies revealed that long‐lived Ames dwarf mice have reduced RQ values when compared to control mice when fed STD (Westbrook *et al*., 2009). We now report that Ames dwarf fed HFD maintain lower RQ values compared to control mice fed either STD or HFD. We also observed that Ames dwarf mice fed STD had lower RQ values compared to control mice fed STD, as expected from our laboratory previous report (Westbrook *et al*., 2009). These data indicate that the long‐lived Ames dwarf mice rely on fat as main source of energy and that HFD feeding does not compromise their ability to maintain a beneficial metabolic profile as seen in Ames dwarf mice fed STD.

Energy expenditure has been linked to metabolic profiles in obese as well as nonobese humans (McGandy *et al*. 1966). In addition, the relationship of energy expenditure to energy intake is reported to be associated to metabolic profiles that predict extended longevity in mice with altered GH signaling or under dietary interventions (Westbrook *et al*., 2009). We now report that Ames dwarf mice fed HFD have increased energy expenditure compared to control mice fed HFD, likely reflecting an increased thermic effect of food intake, resulting in increased calorie burning and locomotor activity. Furthermore, we observed that control mice fed HFD have decreased energy expenditure and locomotor activity compared to control mice fed STD and Ames dwarf mice fed either diet.

### Visceral fat transplant

Surgical removal of visceral fat from laboratory rodents has been shown to improve metabolic function, including insulin sensitivity (Barzilai *et al*., 1999). Furthermore, reduction of visceral fat by omentectomy produced significant positive and long‐term effects on glucose homeostasis in humans (Thorne *et al*., 2002). Ames dwarf mice have increased subcutaneous and visceral adiposity and yet are more insulin sensitive and live much longer than control mice (Brown‐Borg *et al*., 1996; Bartke, 2008)**.** It was recently reported that the removal of visceral adipose tissue from Ames dwarf mice decreased their insulin sensitivity compared to sham‐operated Ames dwarf mice, yet they remained more insulin sensitive than normal mice (Menon *et al*., 2014). In addition, in the same study, removal of visceral fat in Ames dwarf mice decreased the expression of genes related to insulin signaling including, insulin receptor (ir), insulin receptor substrate‐1 (irs‐1), phosphatidylinositol‐3‐kinase (pi3k), protein kinase B (akt2), glucose transporter 4 (glut 4), peroxisome proliferation‐activated receptor‐gamma (ppar‐γ), and peroxisome proliferator‐activated receptor‐gamma coactivator 1 alpha (pgc‐1α) (Menon *et al*., 2014).

These studies suggest that the visceral fat of Ames dwarf mice serves as a ‘beneficial’ contributor to their metabolic flexibility and profile. We show that Ames dwarf mice are protected from the detrimental effects of HFD on metabolic function and have the ability to retain high adiponectin levels, which correlates with their retention of insulin sensitivity. In addition, we observed that energy expenditure is greater in Ames dwarf mice fed HFD compared to control mice fed HFD. Therefore, we interchanged epididymal adipose tissue between full sibling donor Ames dwarf mice fed HFD and control mice fed HFD to assess insulin sensitivity within these groups. Our data shows that surgically transplanting eWAT from Ames dwarf mice fed HFD into control mice fed HFD improved insulin sensitivity and increased adiponectin levels in recipient control mice fed HFD. Hence, transplanting Ames dwarf mouse eWAT into a control recipient mouse fed HFD could rescue it from metabolic dysfunction.

## Conclusion

Excess consumption of diets saturated in fat without sufficient physical activity promotes obesity, metabolic syndrome, and chronic diseases such as type 2 diabetes, cardiovascular diseases, and various types of cancers. The results of the present study indicate that long‐lived hypopituitary Ames dwarf mice are protected from the metabolic dysfunction, but not obesity, caused by HFD. We demonstrate that Ames dwarf mice fed HFD maintain their enhanced insulin sensitivity and exhibit a metabolic flexibility to utilize dietary fat as energy substrate. Furthermore, we conclude that the visceral fat of Ames dwarf mice fed HFD can have a beneficial impact on metabolic characteristics and is able to improve insulin sensitivity in control mice that have been fed HFD for 12 weeks.

## Experimental procedures

### Animals and diet

Ames dwarf mice were produced in our own colony by mating males homozygous for the df (*Prop 1*) mutation (df/df) with heterozygous (df/+) females. Progeny consists of df/df and df/+ animals (the latter are phenotypically normal and were used as normal controls). Animals were entered in the study at 12–14 months of age and were provided ad libitum with nutritionally balanced experimental diets. In experiment 1, four experimental groups consisted of Ames dwarf (*n *= 10) and control (normal) littermate (*n *= 10) males fed a standard diet (10% kcal from fat; (STD)) (SD: D12450K Research Diets, New Brunswick, NJ, USA), and Ames dwarf (*n *= 10) and control (normal) littermate (*n *= 10) males fed a high‐fat diet (HFD) (60% kcal from fat) (HF: D12492 Research Diets, New Brunswick, NJ) for 12 weeks. The animals were housed under temperature‐ and light‐controlled conditions (20–23°C, 12‐h light/12‐h dark cycle). All animal protocols for this study were approved by the Southern Illinois University School of Medicine Laboratory Animal Care and Use Committee.

### Indirect calorimetry

Indirect calorimetry was performed using the AccuScan Instruments, Inc. PhysioScan Metabolic System (Columbus, OH, USA). This system utilizes zirconia and infrared sensors to monitor oxygen (O2) and carbon dioxide (CO2), respectively, inside of respiratory chambers in which individual mice are tested. All comparisons are based on animals studied simultaneously in an effort to minimize the effect of environmental variations and calibration on data. After 24‐h acclamation period, mice were monitored in the metabolic chambers for 24 h with ad libitum access to the assigned diet (STD or HFD) and water. Gas samples were collected and analyzed every 10 min. Output parameters include oxygen consumption (VO_2_ mL kg^−1^ min^−1^), respiratory quotient (RQ VCO2/VO2), and activity shown as total distance traveled (cm). Energy expenditure (kcal/hr/kg) was calculated for each animal.

### Body weight and fat pad weight

Body weights were recorded weekly. White adipose tissue (WAT), subcutaneous and visceral (epididymal and retroperitoneal) tissue, and intrascapular brown adipose tissue (iBAT) were removed and weighed at the end of the study. Normalized adipose tissues weights were calculated by final weight of fat pad/ final whole body weight x100.

### Fasted glucose and homeostatic model assessment

During the 12‐week feeding phase of the experiment, mice were fasted overnight for 16 h weekly to measure fasted blood glucose. Blood was collected by cutting the tip of the tail, and fasted glucose was measured with a glucometer (Presto: AgaMatrix, Salem, NH, USA). The homeostatic model assessment was calculated using the following equation: HOMA‐IR = [fasting plasma glucose (mg dL^−1^) × fasting plasma insulin (mIU mL^−1^)] / 405.

### Glucose tolerance testing

Sixteen‐hour‐fasted mice underwent GTT by i.p. injection with 1 g glucose per kg of BW. Blood glucose levels were measured at 0, 15, 30, 45, 60, and 120 min with a glucometer (Prestp: AgaMatrix) for GTT.

### Insulin sensitivity testing

Nonfasted mice were injected i.p. with 1 IU porcine insulin (Sigma, St. Louis, MO, USA) per kg of BW. Blood glucose levels were measured at 0, 15, 30, and 60 min for ITT. The data for both ITT and GTT are presented as a percentage of baseline glucose.

### Measurements of insulin, IGF‐1, adiponectin, leptin, and interleukin‐6

After 12 weeks of feeding the indicated diets, mice were fasted for 16 h for tissue harvest. Following isoflurane (Butler Animal Health Supply, Dublin, OH, USA) anesthesia, mice were bled by cardiac puncture; blood was mixed with EDTA, followed by centrifugation at 10 000 g for 15 min at 4°C for plasma collection. Plasma insulin levels were determined using Ultra‐Sensitive Rat Insulin Enzyme‐Linked Immunosorbent Assay (ELISA) Kit (Crystal Chem Inc., Downers Grove, IL, USA). Plasma IGF‐1 levels were determined using a Rat/Mouse IGF‐1 ELISA Kit (Immunodiagnostic Systems, Adelaide, Australia). Plasma adiponectin and leptin were determined using a Mouse Adiponectin ELISA Kit (Linco Research, St Charles, MO, USA) and Mouse Leptin ELISA Kit (Crystal Chem Inc), and interleukin‐6 levels were determined using Mouse Interleukin‐6 (IL‐6) ELISA MAX Deluxe Kit (BioLegend, Inc, San Diego, CA, USA), respectively. Protocols for all assays were performed according to the manufacturers’ manuals.

### Lipid profile

Plasma levels of NEFAs were measured via colorimetric assays from Wako Chemicals (Richmond, VA, USA), and triglycerides and cholesterol with kits from Pointe Scientific (Canton, MI, USA) following manufacturer's protocols.

### Fat transfer surgery

Two experimental groups consisted of 12‐ to 14‐month‐old Ames dwarf (*n *= 4) and control (*n *= 4) male mice (from the same breeding pairs) fed HFD for 12 weeks (HF: D12492 Research Diets, New Brunswick, NJ, USA). Ames dwarf and control mice (N) males were subjected to visceral fat transplant (VFT) surgery. All procedures were adapted from previously established protocols (Masternak *et al*., 2012; Menon *et al*., 2014). The animals were anesthetized with ketamine/xylazine and shaved and prepared in a sterile environment. Additionally, animals were supplied with ibuprofen in drinking water starting 2 days before and up to 3 days after the surgery. The epididymal fat pads were removed using blunt dissection through a vertical midline incision. We removed as much epididymal fat as was possible without compromising blood supply to the testes. Each VFT procedure consisted of transplantation of epididymal white adipose tissue (eWAT) from one Ames dwarf mouse into a recipient control (normal) mouse and from the now donor control (normal) mouse to the recipient Ames dwarf mouse. Thus, we interchanged fat pads in littermate pairs of Ames dwarf and control mice. After transplant surgeries mice continued to consume HFD for an additional 4 weeks and insulin sensitivity was measured by ITT at the end of the additional feeding phase. The animals were housed under temperature‐ and light‐controlled conditions (20–23°C, 12‐h light/12‐h dark cycle). All animal protocols for this study were approved by the Southern Illinois University School of Medicine Laboratory Animal Care and Use Committee.

### Statistical analysis

Analyses were performed by two‐way ANOVA test and Student's t‐test between groups when justified. RQ and VO2 measurements were averaged over each hour of the 24‐h period from each group (*n *= 6) and analyzed by two‐factor repeated‐measures analysis of variance. All statistics and graphs were done using Prism 5 (GraphPad Software, San Diego, CA, USA). Alpha was set to 0.05. Values are reported as mean ± standard error of the mean (SEM) throughout the Figures.

## Author contributions

C.M.H. and A.B. were responsible for the study design. C.M.H carried out experiments, data analysis and interpretation, and wrote the manuscript. Y.F., J.M., L.S., M.M., and A.B. contributed to *in vivo* experiments, data interpretation, and edited the manuscript. C.M.H and A.B. were responsible for transplant surgeries of epididymal adipose tissue.

## Funding

Support for this study was provided by grants from the National Institute on Aging (RO1 AG019899 and PO1 AG031376).

## Conflict of interest

There is no potential conflict of interest relevant to this article to report.

## Supporting information


**Fig. S1** (a) Absolute body weight for control mice fed STD. (b) Absolute body weight for control mice fed HFD. (c) Absolute body weight for dwarf mice fed STD. (d) Absolute body weight for dwarf mice fed HFD. (e) Body weight gain percentage for control mice fed STD. (f) Body weight gain percentage for control mice fed HFD. (g) Body weight gain percentage for dwarf mice fed STD. (f) Body weight gain percentage for dwarf mice fed HFD.
**Fig. S2** Absolute adipose tissue (AD) weights of Ames dwarf and control mice fed either STD or HFD.
**Fig. S3** (a) VO_2_ in Ames dwarf and control mice fed STD. (b) VO_2_ in Ames dwarf and control mice fed HFD. (c) VO_2_ in control mice fed either STD or HFD. (d) Twenty‐four‐hour RQ in Ames dwarf and control mice fed either STD or HFD. (e) Twenty‐four‐hour energy expenditure in Ames dwarf and control mice fed either STD or HFD. (f) Day and night locomoter activity.
