# Supplementary figures and images for: Long‐lived hypopituitary Ames dwarf mice are resistant to the detrimental effects of high‐fat diet on metabolic function and energy expenditure
Source: Aging Cell. 2016 Mar 17;15(3):509–21. doi: 10.1111/acel.12467 (PMC4854906; doi:10.1111/acel.12467)

# Supplement figure 1

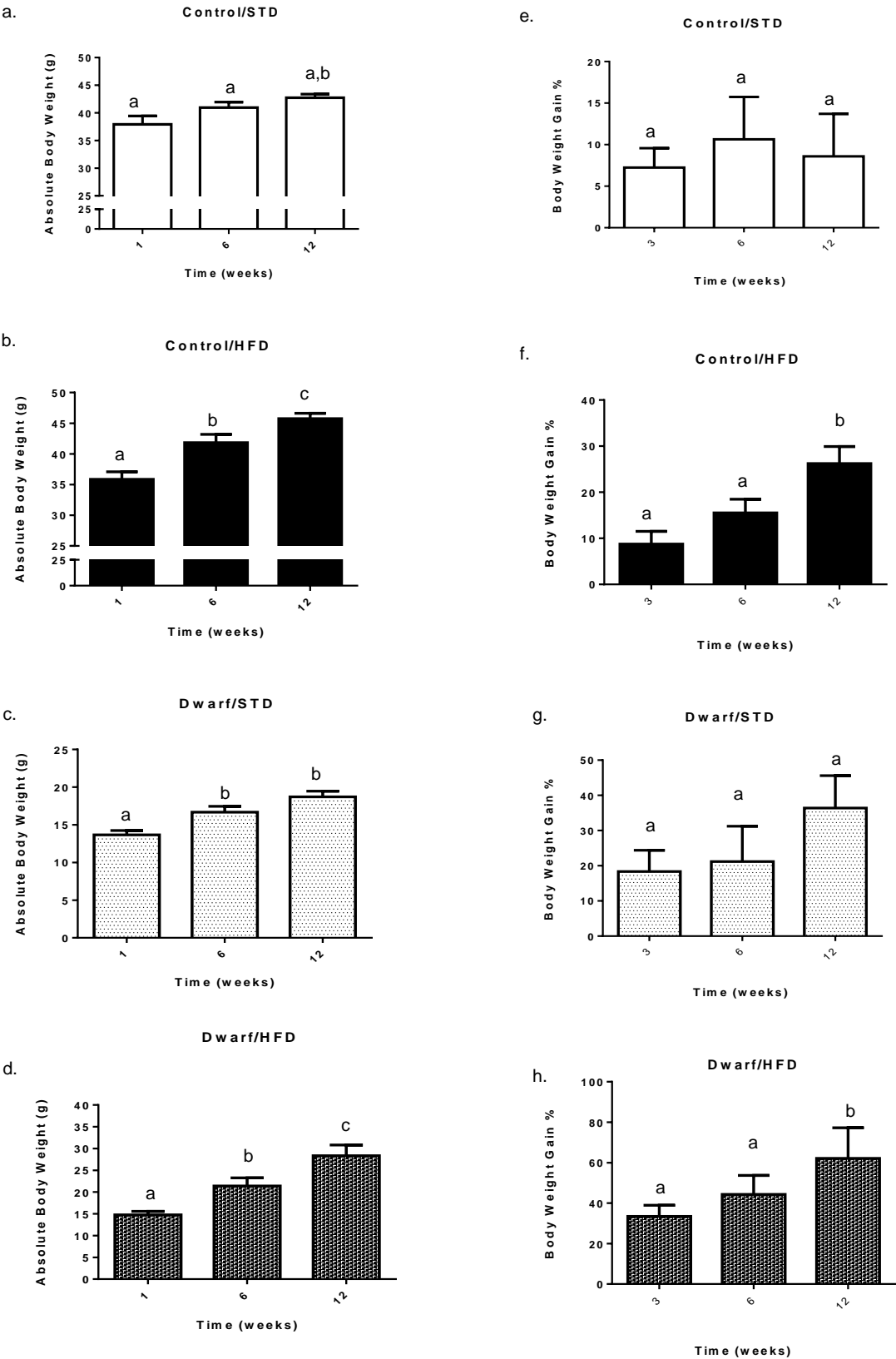

# Supplement figure 2

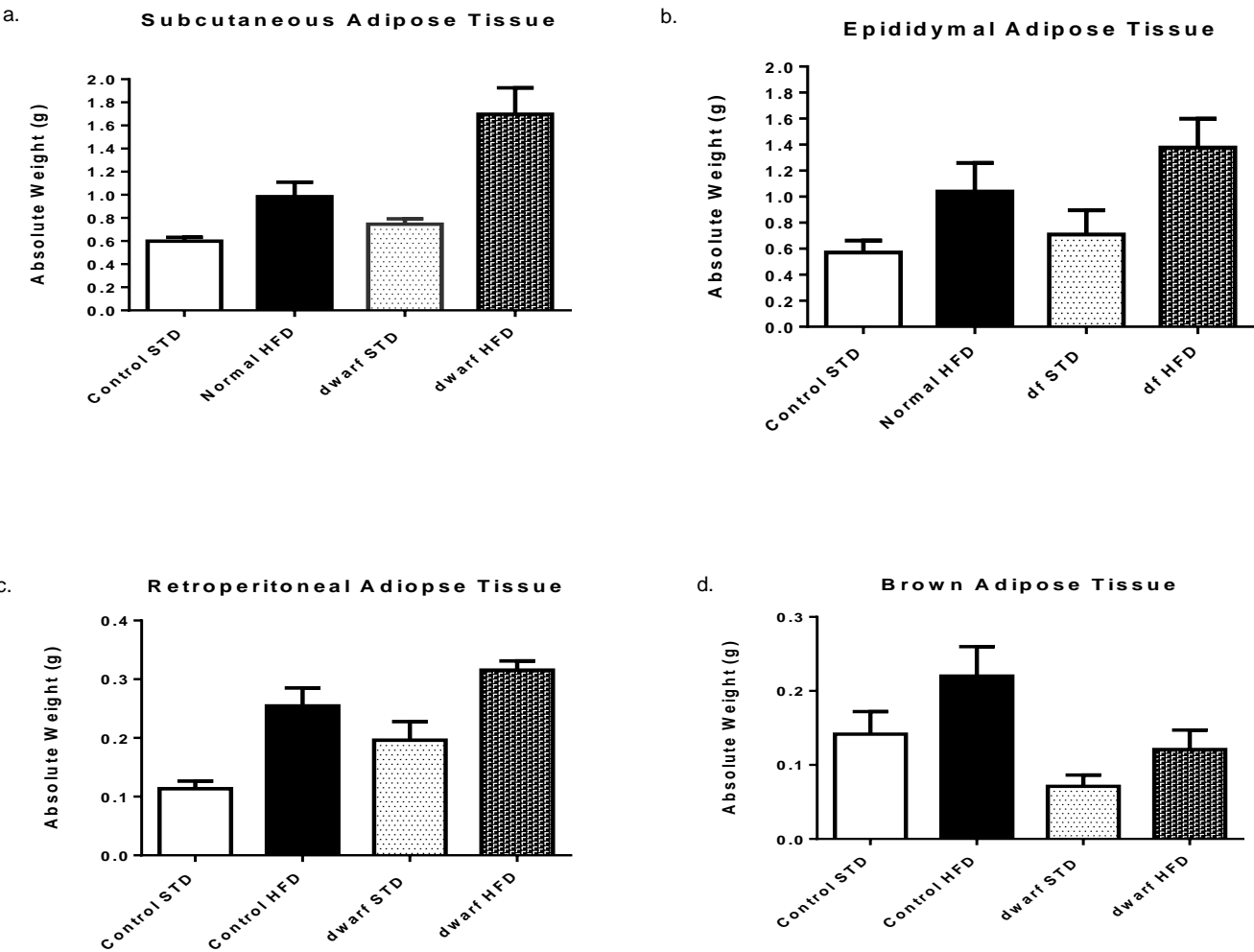

# Supplement figure 3

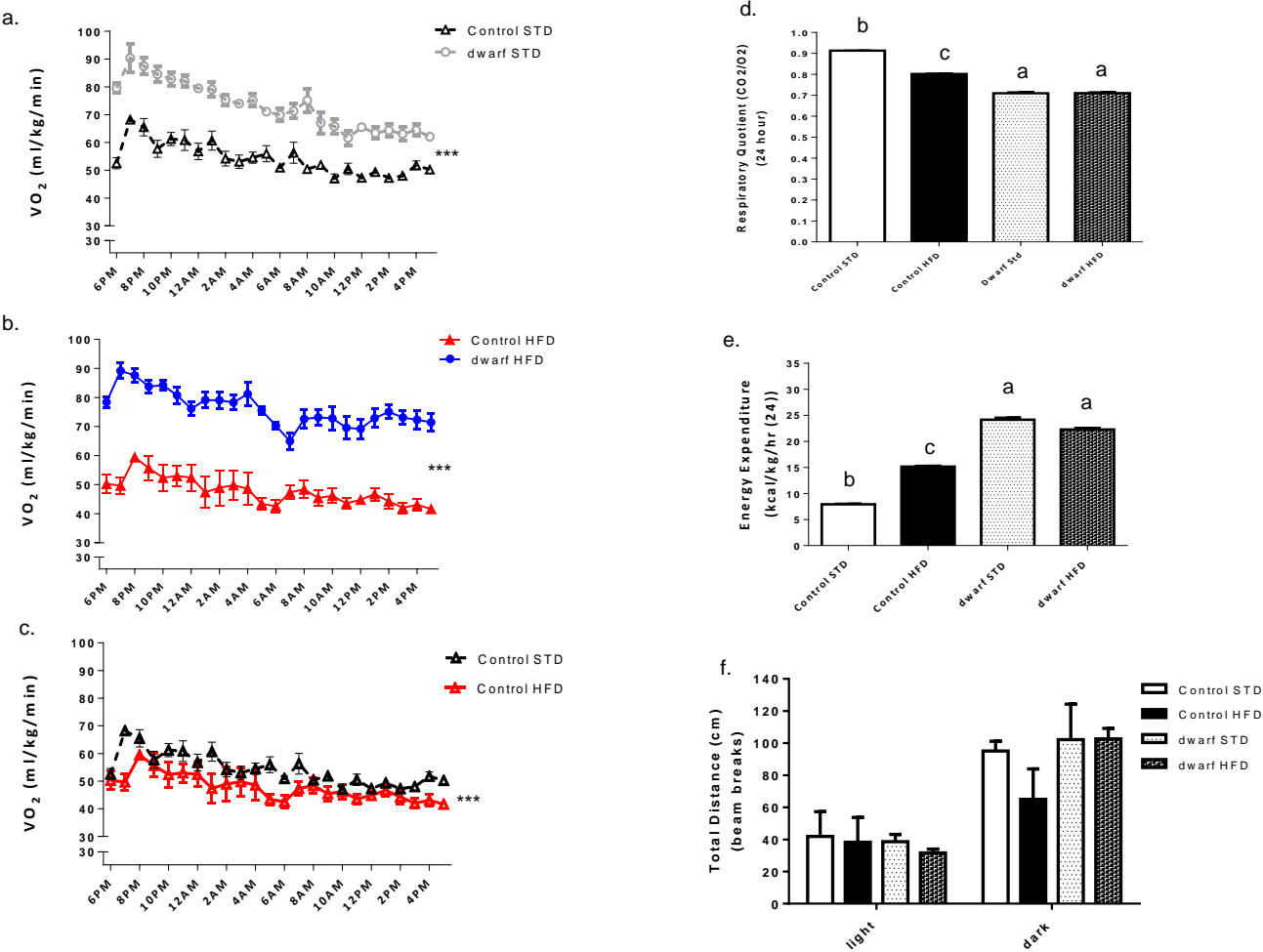

Supplement: Supplementary file 1 — Fig. S1 (a) Absolute body weight for control mice fed STD. (b) Absolute body weight for control mice fed HFD. (c) Absolute body weight for dwarf mice fed STD. (d) Absolute body weight for dwarf mice fed HFD. (e) Body weight gain percentage for control mice fed STD. (f) Body weight gain percentage for control mice fed HFD. (g) Body weight gain percentage for dwarf mice fed STD. (f) Body weight gain percentage for dwarf mice fed HFD. Fig. S2 Absolute adipose tissue (AD) weights of Ames dwarf and control mice fed either STD or HFD. Fig. S3 (a) VO2 in Ames dwarf and control mice fed STD. (b) VO2 in Ames dwarf and control mice fed HFD. (c) VO2 in control mice fed either STD or HFD. (d) Twenty‐four‐hour RQ in Ames dwarf and control mice fed either STD or HFD. (e) Twenty‐four‐hour energy expenditure in Ames dwarf and control mice fed either STD or HFD. (f) Day and night locomoter activity. [file ACEL-15-509-s001.pdf]
